# Case report: Daratumumab for treatment of refractory late or chronic active antibody-mediated rejection in renal allograft recipients with high levels of *de novo* donor-specific antibodies

**DOI:** 10.3389/fimmu.2022.1087597

**Published:** 2023-01-11

**Authors:** Lan Zhu, Zhiliang Guo, Daqiang Zhao, Rula Sa, Guangyuan Zhao, Hui Guo, Gang Chen

**Affiliations:** ^1^ Institute of Organ Transplantation, Tongji Hospital, Tongji Medical College, Huazhong University of Science and Technology, Wuhan, China; ^2^ Key Laboratory of Organ Transplantation, Ministry of Education, Wuhan, China; ^3^ Key Laboratory of Organ Transplantation, National Health Commission (NHC), Wuhan, China; ^4^ Key Laboratory of Organ Transplantation, Chinese Academy of Medical Sciences, Wuhan, China

**Keywords:** kidney transplantation, daratumumab, chronic active antibody-mediated rejection, donor specific antibodies, HLA-DQ, case report

## Abstract

**Background:**

Late or chronic active antibody-mediated rejection (AMR) associated with *de novo* donor-specific antibodies (dnDSA) after renal transplantation is a great clinical challenge because it is often resistant to conventional therapies. Daratumumab, an anti-CD38 monoclonal antibody that can deplete plasma cells, may be effective for the treatment of late or chronic active AMR.

**Methods:**

We designed a novel regimen that included early intensive therapy with daratumumab plus plasmapheresis (PP)/intravenous immunoglobulins (IVIG) and later maintenance therapy with daratumumab alone, and used this regimen to treat late or chronic active AMR in two kidney transplant recipients with extremely high levels of anti-DQ7 dnDSA.

**Results:**

Both patients had a limited clinical response to the early treatment with rituximab and PP/IVIG (with or without splenic irradiation); however, they had a remarkable decrease in anti-DQ7 DSA (MFI value from ~20,000 to ~5,000) after 2-3 months of intensive therapy with daratumumab plus PP/IVIG. Over 20 months of follow-up, patient 1 maintained a low DSA (as low as 1,572) and normal renal function on daratumumab maintenance therapy. Patient 2 retained a low DSA and improved renal function and pathological lesions within one year after treatment but then deteriorated because of acute T cell-mediated rejection.

**Conclusions:**

Our daratumumab-based regimen has shown promising results in the treatment of refractory late active or chronic active AMR in renal transplant recipients with high-level dnDSA. This may provide a reference for better use of daratumumab in the treatment of late or chronic active AMR.

## Introduction

In recent years, late or chronic antibody-mediated rejection (AMR) has been recognized as the major cause of allograft dysfunction and loss in kidney transplantation (1–3). However, late or chronic AMR mediated by *de novo* donor-specific antibodies (dnDSA), even in its active stage, is frequently resistant to the conventional treatments (4–6).

CD38 is a type II transmembrane glycoprotein expressed on various immune cells, and is especially highly expressed on plasma cells and NK cells (7). As an anti-CD38 monoclonal antibody (mAb) initially developed for the treatment of multiple myeloma, daratumumab has been shown to effectively induce complement-dependent cytotoxicity (CDC) and apoptotic signaling in CD38-expressing cells (8, 9). Thus, this antibody may also deplete alloantibody-producing plasma cells, perhaps making it an effective treatment for AMR in organ transplantation.

To date, only a few studies have been published regarding the use of daratumumab for the treatment of AMR or for desensitization in patients awaiting transplantation (10–13). In a reported case of low level of anti-DQ6 dnDSA-mediated chronic active AMR after renal transplantation, 9 months of daratumumab monotherapy resulted in persistent plasma cell depletion, dnDSA disappearance, and stabilization of renal allograft function (10). In another reported case of a combined heart/kidney transplant recipient with severe acute mixed rejection after complete immunosuppressive discontinuation, daratumumab combined with rituximab/eculizumab successfully reduced high levels of anti-DR12 DSA after 8 months of treatment. However, there was no significant change in his anti-DQ7 DSA level (MFI: 20,000~25,000), suggesting that anti-DQ antibodies may be more resistant to daratumumab treatment (12). For chronic active AMR mediated by high levels of anti-DQ dnDSA, which is much more difficult to treat, it is unclear whether daratumumab treatment would also be able to significantly reduce DSA levels and delay the progression of chronic AMR. In the present study, we describe the clinical course and efficacy of a novel daratumumab-based regimen for the treatment of refractory late active or chronic active AMR in two kidney transplant recipients with extremely high levels of anti-DQ dnDSA.

## Materials and methods

### Donor-specific antibody monitoring

Sera were screened for human leukocyte antigen (HLA) antibodies using HLA class I and II single antigen beads (LABScreenTM Single Antigen beads, One Lambda Inc., Canoga Park, CA) as we previously described (14). To avoid antigen saturation, we tested the 1:4, 1:16, 1:32, or 1:64 dilutions of serum samples whose MFI for the 1:3-diluted serum was >15,000. In order to better evaluate the changes of DSA levels detected at different times, a positive serum control was added for each test. The positive control serum was obtained from the residual serum of a highly sensitized patient, and the MFI value of a particular bead was used as a reference for stability in each test.

### The daratumumab-based regimen

The regimen we designed consisted of two phases: an intensive treatment phase and a maintenance treatment phase. In the intensive treatment phase, plasmapheresis (PP)/intravenous immunoglobulins (IVIG, 15-20g each time) plus daratumumab (400-500 mg, intravenously one day after PP/IVIG) was given once a week until the MFI value of the DSA fell to below 5,000. During the maintenance treatment phase, daratumumab alone (400-500 mg, once a week to once a month) was administered intravenously until the DSA disappeared. An intravenous infusion of 5-10 mg dexamethasone and/or an intramuscular injection of 25 mg promethazine was administered before each infusion of daratumumab to prevent an infusion response to daratumumab.

### Compliance with ethics guidelines

The donor kidneys used in both cases were donated to the Red Cross Society and allocated by the China Organ Transplant Response System. The study procedures were approved by the ethics committee of Tongji Hospital, Tongji Medical College, Huazhong University of Science and Technology and performed in accordance with the national program of organ donation in China. The clinical activities being reported are consistent with the principles of the Declaration of Istanbul as outlined in the “Declaration of Istanbul on Organ Trafficking and Transplant Tourism”.

## Results

### Case 1:

**Figure 1:**
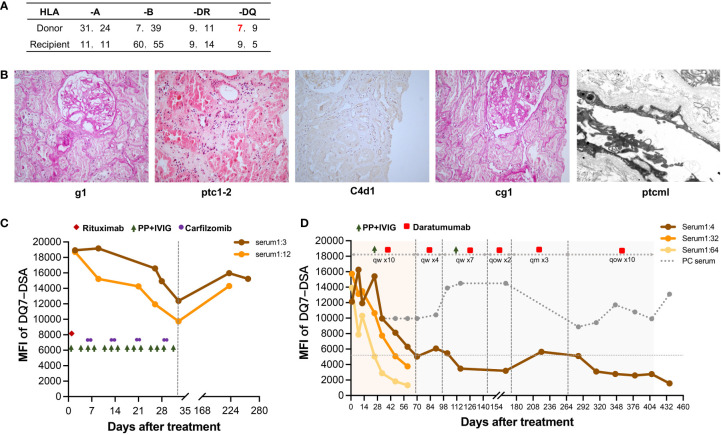
Histological features of subclinical chronic active AMR in Patient 1 before treatment and changes in DSA throughout treatment. **(A)** HLA phenotype results for the recipient and his donor. **(B)** Typical histological findings of chronic active AMR and their Banff 2017 Scores. **(C)** Changes in MFI values of the anti-DQ7 DSA (1:3 or 1:12-diluted serum) during the first round of treatment containing rituximab, PP/IVIG, and carfilzomib. **(D)** Changes in MFI values of the anti-DQ7 DSA (1:4/1:32/1:64-diluted serum and positive control serum) after the treatment with daratumumab-based therapy.

The patient is a 50-year-old man weighing 60 kg who underwent a kidney transplant 5 years ago after one year of hemodialysis. The renal graft was obtained from a 47-year-old brain-dead donor whose cause of death was severe brain trauma. The donor-recipient HLA-A, -B, -DR, -DQ mismatch grade was 6 (**Figure 1A**). The pre-transplant panel-reactive antibody (PRA) and CDC test results were both negative. The patient received induction therapy with anti-CD25 mAb. He recovered smoothly after the operation, and the renal allograft function soon returned to normal. Since the transplant, the patient has received triple maintenance immunosuppressive therapy with oral tacrolimus, mycophenolate mofetil, and prednisone. Flow-PRA test was performed six months after transplantation, and the result was negative.

One and a half years after the transplantation, routine antibody monitoring detected positive Flow-PRA (II: 56.7%) and single antigen beads testing further identified dnDSA against DQ7 with an MFI value of 9,913, at which time the renal graft function was normal. The patient did not receive any specific DSA treatment. After one year of follow-up, the MFI value of anti-DQ7 DSA had increased significantly to 19,167; therefore, graft biopsy was performed. Pathology examination revealed mild glomerulitis (Banff 2017 Schema: g1), mild-to-moderate peritubular capillaritis (ptc1-2), mild peritubular capillary C4d deposition (C4d1), focal glomerular basement membrane double contours (cg1), and peritubular capillary basement membrane multilayering (ptcml, electron microscopy), which were diagnosed as subclinical chronic active AMR (**Figure 1B**). Upon admission, the patient received his first round of treatment for chronic AMR: rituximab (200 mg), carfilzomib (30 mg twice weekly), and 14 PP/IVIG (25g each time) sessions (three times weekly). After 4 weeks of treatment, the MFI level of anti-DQ7 DSA decreased significantly but was still >12,000 (**Figure 1C**).

Six months after the first round of treatment, the MFI value of the anti-DQ7 DSA had rebounded to 15,960 (1:3 diluted serum), whereas the serum creatinine (SCr) level remained stable. After 4 months of follow-up, the MFI value remained high, at 15,237. Given the poor efficacy of conventional therapies in reducing high levels of DQ-DSA, we considered a new daratumumab-based regimen (described above) for this patient. After the intensive treatment with 10 sessions of PP/IVIG plus daratumumab, the MFI of the anti-DQ7 DSA had decreased dramatically, to 5,030 (1:4 diluted serum). When we further diluted the serum samples to 1:32 and 1:64, the DSA-MFI values demonstrated a more obvious reduction (from 15,731 to 3,763 at 1:32 dilution; from 13,926 to 1,320 at 1:64 dilution) (**Figure 1D**). PP/IVIG was then discontinued, but daratumumab was maintained as sequential monotherapy at 400 mg per week. Four weeks later, PP/IVIG was added back in the subsequent treatment because the DSA-MFI value had increased slightly (6,069 at 1:4 serum dilution). After seven weeks of combination therapy, the DSA-MFI value was successfully reduced to 3,475. Therefore, PP/IVIG was discontinued again, and daratumumab was given every 2 weeks for 1 month, followed by monthly administration for 3 months. Since the DSA-MFI value had slightly rebounded, to 5,640, maintenance therapy with daratumumab was again administered biweekly. Seven months later, the DSA-MFI value eventually dropped to 1,572 (**Figure 1D**).

The patient has been on daratumumab therapy for 1 year and 8 months, and currently feels well. He volunteered to continue maintenance treatment until the DSA was completely negative. His renal graft function has remained normal and stable throughout the treatment. At the latest follow-up, he had a SCr level of 83 μmol/L, an eGFR of 95 ml/min/m^2^, and no proteinuria. Histological re-examination was not performed because the patient did not consent to repeat renal graft biopsy. There were no significant adverse effects throughout the treatment, and only mild flu-like symptoms were observed during the initial administration of daratumumab.

### Case 2:

**Figure 2:**
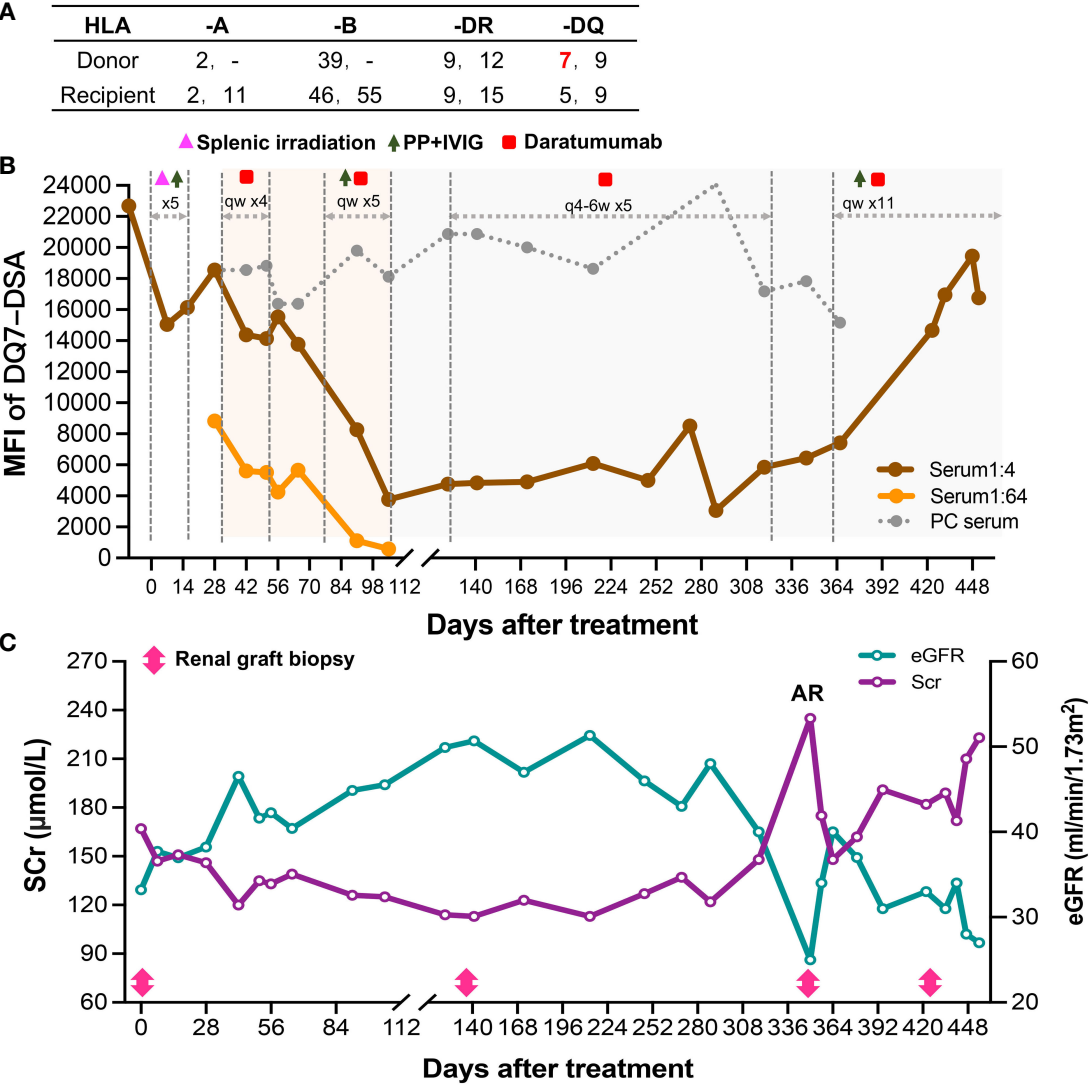
Course of Patient 2’s DSA and renal function after the diagnosis of late active AMR. **(A)** HLA phenotype results for the recipient and his donor. **(B)** Changes in MFI values of the anti-DQ7 DSA (1:4 or 1:64-diluted serum and positive control serum) during the treatment. **(C)** Changes in serum creatinine and eGFR levels after the diagnosis and treatment of late active AMR.

The patient is a 15-year-old male adolescent weighing 38 kg who received a kidney transplant from a 10-year-old deceased donor at a local hospital in May 2019 after 4 months of hemodialysis. The donor-recipient HLA-A, -B, -DR, -DQ mismatch grade was 4 (**Figure 2A**). The pre-transplant PRA and CDC were both negative. Anti-CD25 mAb was used as induction therapy, and a tacrolimus-based triple maintenance immunosuppressive therapy was used to prevent rejection. His SCr decreased to 70 μmol/L at 2 weeks after transplantation and remained stable during early follow-up.

**Figure 3:**
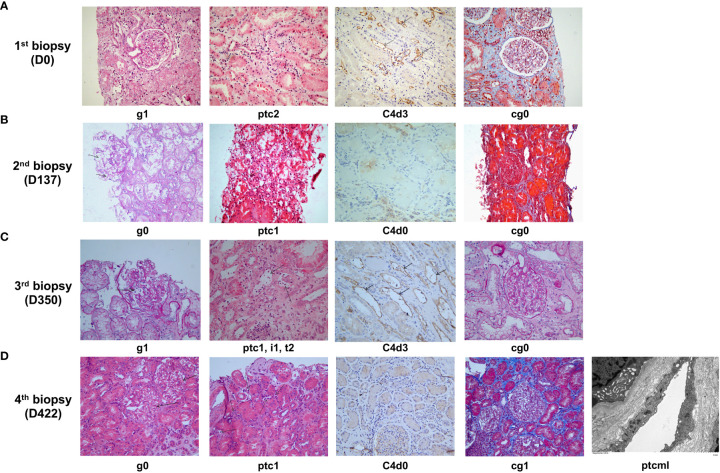
Histological features of late active AMR in Patient 2 before and after treatment. **(A)** Typical histological findings of active AMR in Patient 2 before the treatment and their Banff 2019 Scores. **(B)** The pathology results of a second renal biopsy performed on day 137 after the initiation of treatment and their Banff 2019 Scores. **(C)** The pathology results of a third renal biopsy performed on day 350 after the initiation of treatment (when acute rejection was clinically suspected) and their Banff 2019 Scores. **(D)** The pathology results of a fourth renal biopsy performed on day 422 after the initiation of treatment (when poor response was observed to daratumumab intensive therapy) and their Banff 2019 Scores.

Nineteen months after transplantation, the patient’s SCr level suddenly increased (236 μmol/L), suggesting the possibility of acute rejection. He was immediately admitted to a local hospital and received methylprednisolone (MP) pulse therapy (400 mg/day for 3 days). After the treatment, his SCr decreased to 167 μmol/L but failed to recover further. Meanwhile, anti-DQ7 dnDSA at an MFI value of 8,875 was detected. The patient was referred to our hospital for further treatment. After admission, a biopsy of the renal graft was performed, and pathological findings confirmed active AMR (Banff 2019 Schema: g1, ptc2, C4d3, cg0; **Figure 3A**). We retested the patient’s DSA and found that the MFI value of anti-DQ7 dnDSA had increased further to 22,678. Thus, treatment consisting of rituximab (200 mg), five PP/IVIG (15 g/each time) sessions (every 3-5 days), and splenic irradiation (five times, 50 cGy each (15) was given within an 18-day course. After treatment, the DSA-MFI value had decreased by ~30% (**Figure 2B**). Unfortunately, PP/IVIG and splenic irradiation had to be suspended because of sepsis secondary to the infection associated with internal jugular vein cannulation.

At 15 days after discontinuation of treatment, the MFI value of the anti-DQ7 DSA had rebounded to 18,548. Given the efficacy of daratumumab in reducing DQ-DSA in case 1, we recommended that the patient be treated with daratumumab. With the informed consent from the patient and his parents, daratumumab monotherapy (400 mg, intravenously once a week) was given first. Plasmapheresis was not used concurrently because the neck dialysis cannula had been removed because of infection. After 4 weeks of treatment, the MFI value of anti-DQ7 DSA was reduced by 12%, from 18,548 to 16,374, in the 1:4 diluted serum, and by 50%, from 8,827 to 4,249, in the 1:64 diluted serum (**Figure 2B**). The patient’s renal graft function was simultaneously improved (SCr, 133 μmol/L; eGFR increased from 38 to 46 ml/min/1.73m^2^) (**Figure 2C**). To accelerate the decline in the remaining DSA, PP/IVIG (20 g/each time) was added once a week on the day before the daratumumab infusion. After 5 weeks of combination therapy, the MFI value of anti-DQ7 DSA had decreased markedly, to 3,760 at a serum dilution of 1:4 (**Figure 2B**). Since then, we have withdrawn the PP/IVIG and extended the interval of daratumumab monotherapy to every four weeks. During the first 4 months of the maintenance treatment, the DSA-MFI value of the 1:4-diluted serum remained low, at around 5,000 (**Figure 2B**). To determine whether there was any concomitant histological improvement, a second renal graft biopsy was performed on day 137 after the initiation of treatment. The results showed that the acute tissue injury was alleviated (g0, ptc1, and C4d0) when compared with the first biopsy, and chronic tissue lesions remained minimal (cg0) (**Figure 3B**). After that, the patient received daratumumab (400 mg) every 4-6 weeks. His SCr remained at 113-137 μmol/L, and his eGFR increased to 46-51 ml/min/1.73m^2^.

At the follow-up on day 346 of treatment, a sudden increase in SCr (235 μmol/L) and a low trough level of tacrolimus (3.7 ng/ml) suggested the possibility of acute rejection (**Figure 2C**). The patient was then admitted for urgent renal graft biopsy, which confirmed acute T cell-mediated rejection (TCMR) of grade IA (i1, t2). In addition, pathological features of active AMR reappeared (g1, ptc1, C4d3) (**Figure 3C**). After rescue therapy with pulse MP (400 mg/day for 3 days), his SCr decreased to 148 μmol/L. Because of an increase in the DSA-MFI value (7,419) on day 364, combination treatment with PP/IVIG and daratumumab (once a week) was started again. However, during the 11 weeks of combination therapy, the patient’s DSA-MFI value did not decline as before but continued to rise (to 19,442) (**Figure 2B**). In addition, the patient’s SCr level returned (**Figure 2C**). Therefore, a fourth renal allograft biopsy was performed on day 422. The results showed a few mild areas of microvascular inflammation (g0, ptc1), early double-track sign changes (cg1), and changes in the ptcml (5-7 layers) (**Figure 3D**), suggesting that active AMR had progressed to mild chronic active AMR. At the latest follow-up (455 days after treatment), his DSA-MFI value was 16,759, the SCr level was 223 μmol/L, eGFR was 27 ml/min/1.73m^2^, and total 24-hour urinary protein was 367 mg. In view of the poor response, the patient has stopped receiving the antibody removal therapy. During the early period of daratumumab treatment, the patient had flu-like symptoms on the day of daratumumab use, which could be alleviated by Tylenol administration. There was a mild decrease in white blood cell count (3-4 x 10^9^/L) during the treatment, and no other side effects such as abnormal liver function and increased proteinuria were observed.

## Discussion

In recent years, the role of anti-DQ dnDSAs in renal allograft dysfunction has received increasing attention as they are found to be the predominant dnDSA after transplantation (16–18). In addition, anti-DQ dnDSAs often appear to be more resistant to treatment (15). In this study, we used a novel regimen to treat subclinical chronic active AMR or late active AMR in two kidney transplant recipients with extremely high levels of anti-DQ7 dnDSA. Both patients achieved a remarkable decrease in DSA levels after 2-3 months of intensive therapy with daratumumab plus PP/IVIG. Because both patients had a limited clinical response to the early conventional treatments but showed significant responses to later treatment with daratumumab-based therapy, we suggest that daratumumab may have a superior role in the treatment of late active or chronic active AMR.

One case of daratumumab monotherapy for chronic active AMR (low-level DQ-DSA) after renal transplantation has been reported (10). Although satisfactory results were obtained in this case, it is questionable whether the daratumumab monotherapy regimen would also be effective for late or chronic active AMR associated with high levels of DQ-dnDSA. Aguilera Agudo et al. reported a case of a similar daratumumab monotherapy for late AMR (high-level dnDSA) in heart transplantation (13). Despite clinical and histological improvements in this patient, there was only a mild to moderate reduction in DSA titers after treatment. Therefore, we believe that the daratumumab regimen should be modified to achieve better efficacy for the treatment of high levels of dnDSA-mediated late or chronic active AMR.

Although daratumumab can reduce DSA production by depleting plasma cells, the high levels of DSA already present in the circulation take a long time to gradually decay on their own, so the antibody-lowering effect of daratumumab monotherapy may be difficult to show in the short term. Plasmapheresis can directly remove part of the circulating DSA, and IVIG dampens endogenous antibody rebound after depletion with plasmapheresis. Therefore, in the new protocol used in our study, the combination of daratumumab and PP/IVIG was administered to synergistically reduce the high levels of dnDSA in the first phase, followed by treatment with daratumumab alone to progressively remove the remaining low levels of dnDSA. As a result, we achieved significant results in two patients after short-term combination therapy. During maintenance treatment with daratumumab alone, we found that the administration interval was closely related to the rebound of DSA. When the administration interval was extended to or exceeded every 4 weeks, DSA was prone to rebound, whereas the administration of daratumumab every 2 weeks was relatively safe. Patient 2 had acute TCMR occurred after one year of treatment, which led to a significant rebound of DSA and a progressive decline in renal allograft function. At this time, the patient again received the intensive therapy with daratumumab plus PP/IVIG, which failed to reduce DSA as effectively as before. We don’t know exactly why this happens. We speculate that this may be related to acute rejection. CD4^+^ T cell activation may assist splenic B cells or memory B cells to reactivate, differentiate, and produce antibodies (similar to the process of acute AMR), making DSA removal more difficult with anti-plasma cell therapy plus PP/IVIG. Daratumumab can also act on regulatory cells and reduce them, which may theoretically promote the occurrence of acute cellular rejection (19, 20). The risk of acute cellular rejection with long-term daratumumab treatment warrants further attention in future studies.

Antibody removal therapy was initiated in the subclinical AMR phase of the patient 1 while renal function was still normal. Because his anti-DQ-7 dnDSA showed a significant upward trend during follow-up since its discovery, the progression of subclinical AMR to chronic AMR would be significantly slowed down or even stopped if dnDSA was removed or diminished, thus providing maximum benefit to the patient.

We are aware of some shortcomings and noteworthy aspects of this study: 1) In case 1, the pathological improvement after treatment could not be verified because the patient did not consent to post-treatment allograft biopsy. 2) Case 2 did not receive treatment in strict accordance with the maintenance treatment protocol, which may have affected the final efficacy and prognosis. 3) For economic reasons, the therapeutic single dose of daratumumab administered to our patients was approximately half of the standard dose (16 mg/kg). It is worth paying attention to whether increasing a single dose would achieve a better therapeutic effect. 4) Although intensive treatment with daratumumab combined with PP/IVIG can significantly reduce DSA in the short term, how to better prevent DSA rebound or even completely eliminate DSA in the subsequent maintenance phase and when to stop the treatment still need to be further explored.

To our knowledge, this is the first case report of daratumumab for the treatment of refractory late active or chronic active AMR in renal transplant recipients with high-level dnDSA. Although two cases of effective treatment alone cannot define the effectiveness of a particular therapy, our treatment regimen and ideas may provide a reference for the better use of daratumumab in the treatment of refractory late or chronic active AMR in the future.

## Data availability statement

The original contributions presented in the study are included in the article/supplementary material. Further inquiries can be directed to the corresponding author.

## Ethics statement

The studies involving human participants were reviewed and approved by Tongji Hospital of Tongji Medical College, Huazhong University of Science and Technology. Written informed consent to participate in this study was provided by the participants’ legal guardian/next of kin.

## Author contributions

LZ made contributions in the treatments of subjects and drafting the manuscript; ZG participated in the data collection; DZ participated in the performance of the research; RS participated in the data collection; GZ participated in the performance of the research; HG took responsibility for histological diagnosis and interpretation; GC participated in research design and made substantial revisions to the manuscript. All authors contributed to the article and approved the submitted version.
